# Effects of Individual, Spousal, and Offspring Socioeconomic Status on Mortality Among Elderly People in China

**DOI:** 10.2188/jea.JE20150252

**Published:** 2016-11-05

**Authors:** Lei Yang, Pekka Martikainen, Karri Silventoinen

**Affiliations:** Population Research Unit, Department of Social Research, University of Helsinki, Finland

**Keywords:** socioeconomic status, mortality risk, spousal and offspring’s education, CLHLS, Chinese elderly

## Abstract

**Background:**

The relationship between socio-economic status and health among elderly people has been well studied, but less is known about how spousal or offspring’s education affects mortality, especially in non-Western countries. We investigated these associations using a large sample of Chinese elderly.

**Methods:**

The data came from the Chinese Longitudinal Healthy Longevity Survey (CLHLS) from the years 2005 to 2011 (*n* = 15 355, aged 65–105 years at baseline; 5046 died in 2008, and 2224 died in 2011). Educational attainment, occupational status, and household income per capita were used as indicators of socio-economic status. Spousal and offspring’s education were added into the final models. The Cox proportional hazards model was used to study mortality risk by gender.

**Results:**

Adjusted for age, highly educated males and females had, on average, 29% and 37% lower mortality risk, respectively, than those with a lower education. Particularly among men, this effect was observed among those whose children had intermediate education only. A higher household income was also associated with lower mortality risk among the elderly. Male elderly living with a well-educated spouse (HR 0.79; 95% CI, 0.64–0.99) had a lower mortality risk than those living with a low-educated spouse.

**Conclusions:**

Both the socio-economic status of the individual and the educational level of a co-resident spouse or child are associated with mortality risk in elderly people. The socio-economic position of family members plays an important role in producing health inequality among elderly people.

## INTRODUCTION

The inverse association between socioeconomic status and health has been well established.^1^^–^^5^ For all intents and purposes, people with a higher socioeconomic status have universally been found to have better health as measured on various indicators, such as self-rated health and mortality. However, much less is known about whether the socioeconomic status of the spouse or offspring affects the health and longevity of the partner or parent, respectively. Some studies indicate that spousal and offspring’s education have significant effects on an individual’s mortality, but these were all conducted in high-income countries.^6^^–^^12^ Little is known about how the socio-economic status of other family members affects health in non-Western countries, notwithstanding that the role of family ties and obligations may be more important to the health of elderly in this cultural context.

In contrast to Western societies, in which most elderly people live separately from their adult children, co-residence with family members at old ages is still common in China, where filial piety is still considered to be one of the fundamental values ensuring familial harmony and development.^13^ Most previous studies consider socioeconomic status an individual-level rather than a family-level resource.^14^^–^^16^ Conceptualized as a family-level resource, the health of the elderly depends not only on their own socioeconomic status but also on that of their family members. It is likely that the educational levels of family members will have a stronger association with mortality among the elderly in China than has been observed in other countries because of the commonness of intergenerational co-residence. Several studies have examined the effect of elderly Chinese people’s own socioeconomic status on their mortality risk,^17^^,^^18^ but the extent to which the socioeconomic status of other family members, in particular spouses and children, does or does not affect their mortality is still unclear.

This study investigated whether and to what extent spousal and offspring’s education influences mortality among elderly males and females, net of the individual’s own socioeconomic status. In addition, we examined the interaction effects between the educational levels of elderly people and of their children to assess the joint contribution of these socioeconomic factors on mortality and thereby assess whether a high level of education among offspring can offset the effects of low parental education.

## METHODS

### Data

We used data from the Chinese Longitudinal Healthy Longevity Survey (CLHLS), which was conducted by the Centre for Healthy Aging and Family Studies at Peking University. CLHLS was based on longitudinal survey data gathered via internationally compatible questionnaires from large samples focusing on healthy longevity among the elderly in China. The survey was initiated in 1998 based on a randomly selected sample of older Chinese adults from 22 of the 31 provinces of mainland China, which account for about 85% of the total population of mainland China.^17^ The first two surveys mainly targeted those aged 80 years and over, and the younger elderly (aged 65 years and above) were added from the 2002 wave. The method to select younger elderly was similar to that of selecting those aged 80 years and above. A follow-up face-to-face interview survey was conducted every 2 or 3 years. The survey contained extensive information on Chinese elderly people, including socio-economic position, family structure and background, living arrangements, daily activities, and health condition. Dates of death were validated based on death certificates and confirmation from relatives. We obtained permission from the Centre for Healthy Aging and Family Studies at Peking University to use the data.

Children’s education was added into the survey starting in 2005. We therefore selected the sample of elderly people aged between 65 and 105 years in 2005 as our baseline. The analytical baseline sample used in this study comprised 15 355 respondents. Of these, 5046 died, 2899 were lost to follow-up, and 7410 survived to 2008. By the year 2011, 2224 had died, 1017 had been lost to follow-up, and 4169 survived. Altogether, these respondents yielded 26 935 person-years of records during the nearly 6-year study period.

### Measures

Respondent socioeconomic status was measured using the highest levels of educational attainment, occupational status, and household income per family member. Education was measured in years of schooling in the data. Because nearly half of the elderly had not had any formal education, it was recoded in three categories: low (no schooling, 0 years), intermediate (primary school, 1–6 years), and high (middle school or more, 7 years or more). Occupational status before the age of 60 years was classified into three categories: farmers, white-collar workers (including professional and technical personnel, governmental, institutional or managerial staff, and military personnel), and others. Household income per capita (household total income divided by the number of co-resident family members) was divided into quartiles.

Given the collinearity between spousal education and living arrangements, these variables were recombined into the following categories: 1) low education (0 years), living with a spouse; 2) intermediate education (1–6 years), living with a spouse; 3) high education (7 years or more), living with a spouse; 4) no co-resident spouse.

The co-resident adult children’s education was classified into five categories, which differed slightly from the categories of parental education: a low education included no education and primary school (0 years or 1–6 years), intermediate education included those who attended middle school (7–9 years), and high education indicated upper-secondary education or above (10 years or more). In the case of elderly people living with more than one child, educational attainment reflected the attainment of the most highly educated.

The covariates in this study included residential area (1 = rural area, 0 = urban area); self-rated health (good, fair, or poor); smoking status (current smokers, past smokers, or never smokers); exercise (“Do you exercise regularly at present?”; 1 = yes, 0 = no). Residential area was based on information on the Chinese ‘Hukou’ household registration system; in rural areas, agriculture is an important economic activity, whereas in urban areas, including cities and towns, agriculture is less common. Considering that the proportion of missing values was less than 1% for all variables, those with missing information were categorized separately.

### Statistical methods

We first derived the descriptive statistics and age-adjusted death rates (number of deaths per 10 000 person-years) stratified by gender. We then estimated the multivariate Cox proportional hazards model to study mortality. All the analyses were conducted separately for males and females, given that mortality risk varied by gender. Survival time was calculated in days from the date of the first interview in 2005 to that of the last interview in 2011 for survivors, and to the date of death for the deceased. In the case of those who were lost to follow-up between the different waves, survival time was the number of days from the first interview date in 2005 to the last known interview date.

We estimated four different models for males and females and reported their hazard ratios (HRs) and 95% confidence intervals (CIs) in the tables. Model 0 is an age-adjusted model with each independent variable included separately. The respondents’ socioeconomic-status indicators (education, occupational status, and household income per capita), age, and residential area were included simultaneously in model 1. Next, spousal and offspring’s education were added in model 2 to assess the extent to which the effect of the elderly person’s socioeconomic status on mortality was mediated by the educational level of their spouse or adult children. This approach was used because we found that the individual’s education was associated with all other indicators of socio-economic position (eTable 1). Finally, we included the self-rated health and health-behavior variables (ie, smoking status and exercise) in the final model 3. These variables were considered as mediating variables that are possible on the causal pathway between the socio-economic variables and mortality. We also present the interaction effects between the parent’s and the children’s education in predicting age-adjusted mortality risk. Ethical approval was not required, as this study was a secondary analysis of open-access data and there was no individual identification information in the data. All analyses were performed using Stata 11.2 (Stata Corp, College Station, TX, USA).

## RESULTS

Table 1 presents the descriptive statistics and age-adjusted death rates for males and females. Men were more highly educated than women, with only about 4% of women having a high education. The proportions of male and female farmers were about 56% and 64%, respectively. Overall, the distributions of per capita household income and children’s education were similar among males and females. The varying distributions of education were also reflected in spousal education; for example, 33% of the males had a spouse with a lower level of education, which was almost five times higher than the rate among the spouses of females. Men had higher age-adjusted mortality rates than women for all variables.

**Table 1.** Distributions of all variables and death rate of the sample

	Male (*n* = 11 927)		Female (*n* = 15 008)	
	
	%	Death Rate^a^	%	Death Rate^a^
Education				
low	34.6	2200	80.3	1874
intermediate	46.6	2039	15.1	1936
high	18.5	1961	4.2	1662
missing	0.3	—	0.4	—
Occupation status				
farmers	55.8	2152	64.0	1852
white collar	16.5	1844	3.5	1539
others	27.5	2058	32.2	1909
missing	0.2	—	0.3	—
Household income per capita				
lowest	28.4	1634	31.1	1470
second	19.6	1544	21.2	1255
third	25.0	1255	24.3	1108
highest	26.5	1018	22.8	912
missing	0.5	—	0.6	—
Residential area				
urban	47.0	1263	46.0	1111
rural	53.0	1428	54.0	1284
Spousal education				
low	33.1	1194	7.1	974
intermediate	14.0	1062	9.6	860
high	5.7	1277	3.9	1077
no co-resident spouse	46.7	1430	78.9	1213
missing	0.5	—	0.5	—
Children’s education				
low	13.7	1822	22.3	1419
intermediate	14.2	1612	17.1	1295
high	12.0	1238	15.6	1035
no co-resident children	59.5	1186	44.0	1120
missing	0.6	—	1.0	—
Self-rated health				
good	47.2	1175	41.8	1100
fair	32.5	1360	31.5	1144
poor	20.3	1756	26.6	1440
Smoke status				
current smokers	33.9	1311	6.8	1134
past smokers	31.5	1432	7.6	1291
never smoke	34.6	1306	85.6	1206
Exercises regularly	40	1107	27	995
Number of deaths	3029	—	4241	—

^a^Age-adjusted death rate, per 10 000 person-years.

Table 2 and Table 3 present the HRs from the Cox proportional hazards model predicting mortality among males and females, respectively. Education was inversely associated with mortality risk among both males and females. Among males, those with an intermediate or high education had a nearly 13% lower mortality risk than those with a low education (model 1). A high household income had a significant effect on mortality risk; for instance, the risk of death among elderly men in the third and highest household-income quartiles was 37% and 48% lower, respectively, than among those in the lowest income quartile (model 1). When both spousal and offspring’s education were added in model 2, the effect of education on mortality weakened but remained significant. The effect of household income changed slightly. Spousal and offspring’s education also had a protective effect on reducing older people’s mortality risk: the risk of death among those whose spouse had an intermediate or a high compared to a low education was 20% and 21% lower, respectively (model 2). The HR for those living with children educated to a high level was 0.83 (95% CI, 0.74–0.92) (model 2). When self-rated health and health-related behaviors (ie, smoking and regular exercise) were included in model 3, the effect of the elderly person’s education continued to decline but the HRs of spousal and offspring’s education did not change much (model 3). This suggests that the effects of relative educational variables on mortality are not mediated through self-rated health and health-related behaviors.

**Table 2.** Hazard ratios and 95% confidence intervals from Cox proportional model for males (*n* = 11 927)

	Model 0^a^	Model 1	Model 2	Model 3

	HR (95% CI)	HR (95% CI)	HR (95% CI)	HR (95% CI)
Education (ref. = low)				
intermediate	0.81 (0.76–0.87)	0.86 (0.80–0.92)	0.87 (0.81–0.93)	0.88 (0.82–0.94)
high	0.71 (0.64–0.78)	0.87 (0.78–0.97)	0.90 (0.80–1.00)	0.94 (0.84–1.05)
missing	0.88 (0.52–1.49)	1.05 (0.59–1.89)	1.08 (0.60–1.95)	1.06 (0.59–1.91)
Occupation status (ref. = farmers)				
white collar	0.72 (0.65–0.79)	1.03 (0.92–1.15)	1.11 (0.99–1.25)	1.14 (1.01–1.27)
others	0.83 (0.77–0.89)	1.08 (1.00–1.18)	1.10 (1.02–1.19)	1.12 (1.03–1.21)
missing	0.82 (0.39–1.72)	0.84 (0.37–1.93)	0.82 (0.36–1.87)	0.82 (0.36–1.86)
Household income per capita (ref. = lowest quartile)				
second quartile	0.84 (0.77–0.91)	0.86 (0.79–0.93)	0.83 (0.77–0.91)	0.85 (0.78–0.92)
third quartile	0.60 (0.55–0.66)	0.63 (0.58–0.69)	0.64 (0.58–0.70)	0.65 (0.60–0.71)
highest quartile	0.48 (0.44–0.52)	0.52 (0.47–0.57)	0.54 (0.49–0.59)	0.55 (0.50–0.60)
missing	0.18 (0.08–0.41)	0.19 (0.09–0.43)	0.19 (0.08–0.43)	0.23 (0.10–0.52)
Age, years	—	1.07 (1.06–1.07)	1.06 (1.05–1.06)	1.06 (1.05–1.06)
Rural area (ref. = urban)	1.41 (1.32–1.51)	1.19 (1.12–1.29)	1.16 (1.08–1.25)	1.14 (1.06–1.23)
Spousal education (ref. = low)				
intermediate	0.68 (0.60–0.78)		0.80 (0.69–0.91)	0.81 (0.70–0.92)
high	0.58 (0.47–0.72)		0.79 (0.62–0.97)	0.79 (0.64–0.99)
no co-resident spouse	1.17 (1.08–1.26)		1.06 (0.97–1.14)	1.06 (0.98–1.15)
missing	1.56 (1.03–2.35)		1.83 (1.21–2.77)	1.87 (1.23–2.82)
Children’s education (ref. = low)				
intermediate	0.89 (0.80–0.99)		0.97 (0.87–1.07)	0.98 (0.89–1.09)
high	0.70 (0.63–0.78)		0.83 (0.74–0.92)	0.85 (0.76–0.95)
no co-resident children	0.56 (0.52–0.61)		0.65 (0.60–0.71)	0.66 (0.60–0.72)
missing	0.34 (0.22–0.54)		0.42 (0.27–0.65)	0.43 (0.28–0.68)
Self-rated health (ref. = good)				
fair	1.11 (1.03–1.19)			1.08 (1.00–1.16)
poor	1.49 (1.38–1.61)			1.37 (1.27–1.48)
Smoke status (ref. = current smokers)				
past smokers	1.04 (0.96–1.12)			1.09 (1.01–1.18)
never smoke	0.91 (0.84–0.98)			0.94 (0.87–1.02)
Exercises regularly	0.68 (0.63–0.73)			0.80 (0.74–0.85)
-Likelihood	—	35 601.74	35 505.46	35 434.34

CI, confidence interval; HR, hazard ratio.

^a^Age adjusted model with each variable.

**Table 3.** Hazard ratios and 95% confidence intervals from Cox proportional model for females (*n* = 15 008)

	Model 0^a^	Model 1	Model 2	Model 3

	HR (95% CI)	HR (95% CI)	HR (95% CI)	HR (95% CI)
Education (ref. = low)				
intermediate	0.84 (0.77–0.91)	0.91 (0.83–0.99)	0.92 (0.84–1.01)	0.94 (0.86–1.03)
high	0.63 (0.52–0.75)	0.83 (0.68–1.01)	0.87 (0.71–1.07)	0.92 (0.75–1.12)
missing	0.79 (0.53–1.18)	0.73 (0.44–1.20)	0.71 (0.43–1.18)	0.72 (0.43–1.18)
Occupation status (ref. = farmers)				
white collar	0.64 (0.52–0.78)	0.97 (0.78–1.20)	0.98 (0.79–1.22)	0.99 (0.80–1.23)
others	0.91 (0.86–0.97)	1.16 (1.09–1.24)	1.17 (1.09–1.24)	1.17 (1.09–1.25)
missing	0.99 (0.60–1.64)	1.47 (0.78–2.77)	1.46 (0.77–2.77)	1.56 (0.82–2.95)
Household income per capita (ref. = lowest quartile)				
second quartile	0.79 (0.73–0.84)	0.80 (0.75–0.86)	0.81 (0.75–0.86)	0.82 (0.76–0.88)
third quartile	0.67 (0.62–0.72)	0.70 (0.65–0.75)	0.71 (0.66–0.77)	0.73 (0.68–0.79)
highest quartile	0.52 (0.48–0.57)	0.56 (0.52–0.61)	0.59 (0.54–0.64)	0.60 (0.55–0.65)
missing	0.09 (0.04–0.22)	0.10 (0.04–0.23)	0.10 (0.04–0.23)	0.14 (0.06–0.33)
Age, years	—	1.07 (1.06–1.07)	1.07 (1.06–1.07)	1.06 (1.06–1.07)
Rural area (ref. = urban)	1.40 (1.32–1.48)	1.28 (1.20–1.36)	1.25 (1.17–1.33)	1.24 (1.18–1.33)
Spousal education (ref. = low)				
intermediate	0.81 (0.66–0.98)		0.84 (0.69–1.02)	0.85 (0.70–1.03)
high	0.66 (0.50–0.88)		0.82 (0.61–1.09)	0.84 (0.63–1.12)
no co-resident spouse	1.17 (1.01–1.36)		1.13 (0.99–1.31)	1.15 (0.99–1.33)
missing	0.83 (0.48–1.41)		1.50 (0.87–2.60)	1.75 (1.02–3.02)
Children’s education (ref. = low)				
intermediate	0.92 (0.85–0.99)		0.98 (0.90–1.05)	0.98 (0.90–1.05)
high	0.69 (0.64–0.76)		0.81 (0.74–0.88)	0.83 (0.76–0.90)
no co-resident children	0.74 (0.69–0.81)		0.79 (0.74–0.85)	0.80 (0.75–0.86)
missing	0.38 (0.28–0.52)		0.50 (0.37–0.68)	0.50 (0.36–0.67)
Self-rated health (ref. = good)				
fair	1.01 (0.95–1.08)			0.96 (0.90–1.03)
poor	1.33 (1.25–1.42)			1.24 (1.16–1.32)
Smoke status (ref. = current smokers)				
past smokers	1.06 (0.92–1.22)			1.05 (0.92–1.21)
never smoke	1.04 (0.93–1.16)			1.01 (0.91–1.12)
Exercises regularly	0.70 (0.65–0.75)			0.79 (0.73–0.85)
-Likelihood	—	49 583.65	49 529.86	49 430.99

CI, confidence interval; HR, hazard ratio.

^a^Age adjusted model with each variable.

The effect of education on mortality risk was slightly different among females than males. Females with an intermediate education had a 9% lower risk of death than those with a low education, but the differences between a high and a low education were not significant (model 1). When spousal and offspring’s education were added in model 2, the effects of educational level declined and became statistically non-significant. Among males, a higher household income reduced the mortality risk of the elderly, which nevertheless remained statistically significant. Net of their own education, those whose co-resident children had a high level of education had a 19% lower mortality risk compared to a low education level (model 2). When all the covariates were added in the final model, the effects of education and occupational status remained non-significant, whereas the effect of household income was still significant.

Table 4 shows the extent to which the results from the interaction effects between the elderly parent’s education and their children’s education predicted mortality for men and women separately. Older males with a low education faced a 31% lower risk of death if their co-resident child had a high education. Among men with a high education, the HR was 0.70 (95% CI, 0.57–0.85) if their co-resident children had a high level of education. Of note, elderly men whose child had low education had 13% higher mortality and elderly women had 8% higher mortality if elderly participants had high education compared to low education (ie, the association between an individual’s own education and mortality was reversed compared to the other categories of children’s education). The interaction effect was statistically significant only for males (*P* = 0.002 for males and *P* = 0.15 for females).

**Table 4.** Interaction effects between parent’s education and children’s education for predicting hazard ratios

Male	HR (95% CI)			HR (95% CI)
		
	Children’s education			Parents’ main-effect
		
Parents’ education	Low	Intermediate	High	
Low	1.00	1.08 (0.93–1.25)	0.69 (0.57–0.82)	1.00
Intermediate	0.88 (0.77–1.02)	0.76 (0.66–0.88)	0.65 (0.56–0.76)	0.82 (0.77–0.88)
High	1.13 (0.89–1.44)	0.62 (0.48–0.80)	0.70 (0.57–0.85)	0.75 (0.68–0.83)
Children’s main-effect	1.00	0.92 (0.83–1.02)	0.73 (0.66–0.81)	
*P* = 0.002				


Female	HR (95% CI)			HR (95% CI)
		
	Children’s education			
		
Parents’ education	Low	Intermediate	High	Parents’ main-effect
Low	1.00	0.92 (0.85–1.00)	0.70 (0.64–0.77)	1.00
Intermediate	0.92 (0.76–1.10)	0.83 (0.69–1.00)	0.65 (0.54–0.79)	0.86 (0.78–0.94)
High	1.08 (0.61–1.91)	0.81 (0.50–1.32)	0.62 (0.45–0.85)	0.70 (0.58–0.84)
Children’s main-effect	1.00	0.92 (0.85–1.00)	0.71 (0.65–0.75)	
*P* = 0.15				

CI, confidence interval; HR, hazard ratio.

All models adjusted for age.

The results of other categories of parent’s education and children’s education were not shown in the table.

## DISCUSSION

Consistent with findings from previous studies, our results confirm the strong association between higher household income and mortality among the elderly.^19^^–^^21^ Economic resources, such as family income, consistently and significantly affect mortality risk in older people in China. Higher household income also predicted a lower mortality risk after adjustment for other covariates, such as the individual’s own socioeconomic status and health-related behaviors. Enhanced economic resources could give the elderly access to a better quality of life and adequate medical care and services.^22^^,^^23^ The effect of income on mortality among the older people investigated in this study turned out to be stronger than has been observed in studies among the elderly in other high-income countries.^7^^,^^24^^–^^26^

Most significantly, our results indicate an association between higher offspring’s education and a lower mortality risk among older people, and this association was especially strong among elderly males. Simultaneous adjustment for an individual’s own socioeconomic status or that of their offspring partly attenuated these effects. We also found that elderly with higher education and with highly educated children had lower mortality. However, the protective effects of higher education tended to be most pronounced among elderly who had children with intermediate-level education, particularly among elderly men. Because of these interactions, the main effect of an individual’s own education on male mortality should be interpreted with caution, as its effects may vary according to the education of co-resident children. Overall, our results indicate that it is not only the individual’s own socioeconomic status that affects health in older people, but also the educational level of the individual’s spouse and offspring. This suggests that, to some extent, education is a household-level rather than a purely individual-level resource.^27^ For both males and females, those who had no co-resident children had lower hazard ratios of mortality. We interpret this to be the result of health selection, in which the healthiest elderly person can live alone, but those who need help with their daily tasks are more likely to live with their children to get care.

Marriage also had a protective effect on health. Elderly people living with a spouse had a lower mortality risk than those without a spouse, and those living with a highly educated spouse had lower risk than those with a spouse with only a basic education. For example, among the males, those living with a highly educated spouse had a 21% lower mortality risk in the fully adjusted model. Our results are consistent with previous findings from England, Sweden, Norway, and Israel indicating that one’s partner’s education is significant as a predictor of one’s own mortality risk.^6^^,^^8^^,^^28^^,^^29^ One possible explanation is that married men and women can share economic resources and give one another social and emotional support.^9^^,^^30^ High education among the women lowered the mortality risk among their husbands more significantly (*P* < 0.05) than vice versa. The fact that highly educated women tend to show better health-related and lifestyle behaviors that benefit the health of their husbands might explain this effect.^9^^,^^28^ However, there is still a need for further investigation in the Chinese context.

Having highly educated adult children is consistently associated with a lower risk of parental death in welfare states, such as the Nordic countries,^11^^,^^12^ where social services for the elderly are strongly supported and most adult children do not live with their parents. We demonstrated a similar association in China, where socioeconomic disparity is increasing, public services for the elderly are moderate, and co-residence with adult children is common. We found in our study that elderly males and females living with a highly educated child had a roughly 15% lower mortality risk than those living with a child educated to a low level. A Swedish study found that people living with a child educated to the tertiary level had a similarly lower mortality risk as those whose co-resident child received only compulsory education.^11^ Although social policy is comparatively egalitarian in welfare societies, and governments support equality in the provision of public healthcare to the elderly, upward intergenerational exchange and support remain strong, and offspring’s education still has a strong effect on their parents’ health.^11^^,^^31^ In China, on the other hand, where government can only supply basic healthcare to increasing numbers of older people, children take the main responsibility for the care of their ill and aging parents, especially in rural areas.^32^^,^^33^ We found that spousal and offspring’s education are equally important for the health of elderly people. From the perspective of policy, efforts to increase the educational level of all people in the future may help to improve health and reduce the mortality of the elderly and reduce health inequality in the long run.

We also found that parents with a higher education tended to benefit more from their highly educated children, which is consistent with the results of earlier research conducted in Taiwan.^27^ Children or other family members still seem to be the main organizers, suppliers, and financiers of healthcare for the elderly in Chinese societies, in which family values and responsibility are highly respected. Highly educated children can afford better medical care and services and have better access to health-related knowledge, to the benefit of their parents’ health.^11^^,^^23^ Highly educated elderly parents living with highly educated children may more readily take advantage of the resources they contribute than old people with a low level of education.^27^

Limitations of this study should also be noted. The first is that we only had sufficient power to analyze overall mortality risk by gender. Some previous studies indicate that spousal education has a different effect on cardiovascular disease (CVD) mortality than on other types of cause-specific mortality,^8^^,^^34^ but we could not analyze this because of the limited data. Another concern is that the measurements of health and health-related behaviors may be inaccurate, and we also lack information on health-related behaviors among family members. Older people’s health may be affected by health-related behaviors, such as smoking; highly educated spouses and children tend to be less likely to smoke, which could influence their co-resident partner’s or parent’s health behavior.

Overall, the socioeconomic resources of family members play an important role in producing health inequality among elderly people. Our results, obtained from analysis of extensive and representative longitudinal data in China, provide strong evidence of an effect of spousal and offspring’s education on partners’ and parental mortality risk, respectively. Our findings indicate that a higher education among family members plays a significant role—in addition to individual socioeconomic resources—in reducing elderly people’s mortality risk. Hence, enhancing the socioeconomic status of offspring may help to reduce socioeconomic differentials and inequality in health and mortality among elderly people in the future.

## ONLINE ONLY MATERIAL

eTable 1. Associations between an individual’s own education, other socioeconomic status, high spousal education, and high children’s education at baseline.
