# Supplementary material for: Effects of Individual, Spousal, and Offspring Socioeconomic Status on Mortality Among Elderly People in China
Source: J Epidemiol. 2016 Nov 5;26(11):602–9. doi: 10.2188/jea.JE20150252 (PMC5083324; doi:10.2188/jea.JE20150252)
Supplement: eTable 1. [file je-26-602-s001.pdf]

eTable 1. Associations between an individual's own education, other socioeconomic status, high spousal education, and high children's education at baseline

**Male**	Highest household income (%)	White collar (%)	High spousal education (%)	High children's education (%)
Own education				
low	27.4	7.0	4.0	21.7
intermediate	42.4	31.7	18.3	45.6
high	29.8	61.0	77.7	32.3
missing	0.4	0.3	0	0.4
n	1,684	1,099	856	792
Total %	100	100	100	100
p value[a]	<0.0001	<0.0001	<0.0001	<0.0001
**Female**				
Own education				
low	69.9	17.3	29.5	66.0
intermediate	18.0	23.9	37.1	23.9
high	11.5	58.8	33.4	9.9
missing	0.6	0	0	0.2
n	1,997	306	326	1,076
Total	100	100	100	100
p value[a]	<0.0001	<0.0001	<0.0001	<0.0001

[a] *p* value is calculated based on chi-square test
